# Mantle cell lymphoma of the larynx: Primary case report

**DOI:** 10.1186/1752-1947-6-201

**Published:** 2012-07-16

**Authors:** Sarah Naciri, Anass A Bennani-Baiti, Meriem Glaoui, Houda Mouzount, Samia Ghanem, Leila Essakali, Mohamed Kzadri, Hassan Errihani

**Affiliations:** 1 Medical Oncology Department, National Institute of Oncology, University Mohamed V, University Hospital, Rabat, Morocco; 2 Oto-Rhino-Laryngology, Head and Neck Surgery Department, Hopital des Spécialités, University Mohamed V, University Hospital, Rabat, Morocco

## Abstract

**Introduction:**

Primary laryngeal lymphomas are exceedingly rare. Only about a hundred cases have been reported. They consist mainly of non-Hodgkin lymphoma, especially of diffuse large B-cell lymphoma and mucosa-associated lymphoid tissue. We report the first case of a primary laryngeal mantle cell lymphoma.

**Case presentation:**

We report a case of a primary mantle cell lymphoma of the larynx in a 70-year-old North African non-smoker male. We present a detailed report of his clinical and paraclinical data as well as treatment options.

**Conclusions:**

Mantle cell lymphoma is a very aggressive lymphoma subset associated with poor prognosis. Laryngeal mantle cell lymphoma is exceedingly rare. To the best of our knowledge, this is the first case to ever be reported.

## Introduction

Primary laryngeal lymphoma is extremely rare, accounting for less than 1% of all primary laryngeal neoplasms [1]. Fewer than 100 cases have been reported in the literature [1]. They consist mainly of non-Hodgkin lymphomas (NHLs) and are predominantly located in the supraglottic region, as this area of the larynx contains follicular lymphoid tissue [2]. Among the subtypes of NHL, diffuse large B-cell and mucosa-associated lymphoid tissue lymphomas are the most commonly encountered primary laryngeal hematopoietic neoplasms [1]. To the best of our knowledge, no prior case of mantle cell lymphoma (MCL) of the larynx has been reported; this subtype represents only 6% of lymphomas, regardless of the localization [3,4]. We report a case of primary MCL of the larynx in a 70-year-old male, with a review of the main features of this subset of extranodal lymphoma.

## Case presentation

We report the case of a 70-year-old North African man who, two months prior to medical examination, presented with mild laryngeal respiratory distress that worsened until he ultimately required a tracheotomy. A direct laryngoscopy had been performed, revealing a subglottic, submucosal lump. A biopsy revealed a mantle cell type B NHL with the following antigen constellation: cluster of differentiation (CD)5+, CD20+, CD23-, cyclin D1+. Importantly, a physical examination and computed tomography (CT) scans showed a lack of lymph node involvement, establishing the larynx as the primary site of the neoplastic lesion.

**Figure 1:**
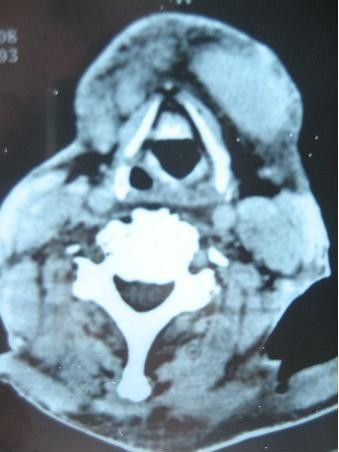
Computed tomography scan showing the upper glottis area free of any tumor.

**Figure 2:**
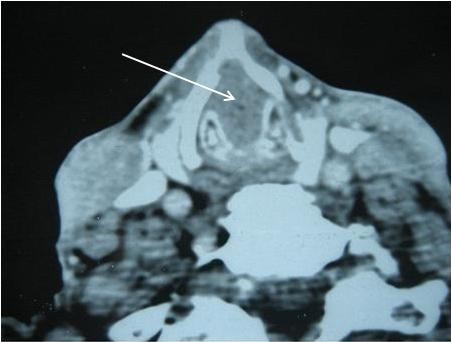
** Computed tomography scan showing a subglottic tumor completely obstructing the laryngeal lumen.** Arrow shows the subglottic tumor while the glottis area is free of disease (Figure 1).

The patient’s history and physical examination found no comorbidities. The patient did not present any B symptoms and had a performance status 3 as measured using the World Health Organization performance scale. During a follow-up four weeks after his initial admission, a physical examination found laterocervical lymph nodes. An investigation into possible tumoral extension by cervical, thoracoabdominal and pelvic CT scans showed a subglottic tumor (Figures 1 and 2) and cervical lymph nodes.

A bone marrow biopsy did not show any anomalies. A blood workup showed he had normal red blood cell sedimentation rate and lactate dehydrogenase levels.

The patient, who has been staged IIEAa (Ann Arbor Classification), received one course of rituximab-cyclophosphamide, doxorubicin, vincristine and prednisone but unfortunately died of unknown causes. No autopsy was performed in compliance with the family’s wish.

## Discussion

Extranodal NHL accounts for 20 to 30% of all lymphomas, while MCL represents only 6% of all NHL subtypes, both nodal and extranodal [3,4]. Laryngeal NHL is exceedingly rare, with very few published cases; to the best of our knowledge, we report the first case of a laryngeal MCL.

Most lymphomas involving the larynx involve other sites as well, including the salivary glands, the thyroid, the nasopharynx and tonsils [1]. The most common site of primary laryngeal lymphomas is the supraglottic region, as it contains lymphoid collections in the lamina propria and in the ventricles [2]. In this case, the tumor localized in the subglottic region of the larynx and involved no other sites as assessed by a thorough ear, nose and throat examination and CT scans.

The age of onset for laryngeal lymphomas varies between 4 and 81 years, with the mean age of occurrence being in the seventh decade (the patient was 70 years old); the distribution between males and females is similar [5].

The symptoms at presentation are common to other laryngeal neoplasms, i.e. hoarseness, dysphonia, dysphagia, stridor, cough and other systemic symptoms, such as weight loss and fever [5]. In some cases, the disease is revealed by a threatening dyspnea requiring an urgent tracheotomy as it was the case with this patient. The mechanical airway obstruction explains the obvious similarity with laryngeal squamous cell carcinoma. Primary laryngeal lymphomas constitute a diagnostic challenge because they are characterized by the absence of clinical and gross differential criteria, as compared to squamous cell carcinoma.

A survey of primary lymphomas of the larynx published from 1996 to 2008 shows that 47% were located in the supraglottic region, 25% in the glottic area, and the remainder were either subglottic or transglottic [6].

Regarding the macroscopic characteristics of the reported cases, 20 of 36 cases (55%) were described either as smooth or submucosal masses, whereas only two cases (5.5%) were ulcerated lesions [6]. While imaging techniques such as CT and magnetic resonance imaging may be helpful in the assessment of any laryngeal neoplasm [7], a definitive diagnosis requires histological examination of a biopsy specimen.

A wide spectrum of histological subtypes of laryngeal lymphomas has been reported. The great majority of laryngeal NHLs are of B-cell lineage, frequently presenting as diffuse large cell lymphoma; very few are of T-cell lineage [8,9]. The malignant cell type of classic MCL is composed of small- to medium-sized lymphocytes with irregular nuclei and condensed chromatin, though a broad spectrum of morphologic features ranging from small cell to blastoid types also exists, and these may reflect distinct biologic characteristics. The immunophenotype of MCL corresponds to mature, naive pre-germinal center B-cells that express the antigens CD19, CD20, CD22, CD79A, immunoglobulin M and/or immunoglobulin D. They usually are CD5+ and CD43+ but CD10- and CD23-. Cyclin D1 overexpression allows, if there were still any doubt, to confirm the diagnosis [10,11]. Immunohistochemistry on the patient’s biopsy specimen revealed that it was CD5+, CD20+, CD23- and cyclin D1+.

While there is not a consensus regarding the treatment of MCL, localized stage I and II patients are usually treated with chemotherapy and radiotherapy, while those with disseminated forms (stages III and IV) are treated with chemotherapy only. MCL is an aggressive lymphoma with the poorest long-term survival of any subtype. This might be linked to the fact that they usually are discovered at an advanced stage. Remission induction using intensive chemotherapy regimens appears to increase the time to progression; it does not, however, improve the overall survival [10,11]. Randomized data have shown that chemotherapy remission rates can be improved by the addition of rituximab [12-14]. Rituximab also appears to improve clinical and molecular responses in patients when used for post-transplant consolidation [15,16]. Randomized data have shown the benefit of autologous stem cell transplantation after high-dose therapy for patients in first remission. Compared with chemotherapy alone, there is improved progression-free survival, although an advantage in the overall survival has yet to be demonstrated [17]. Both myeloablative and non-myeloablative allogeneic transplants have been evaluated as another promising treatment option, which will need further corroboration in larger cohorts. Some newer targeted therapies (for example, bortezomib, thalidomide, temsirolimus, flavopiridol) that have shown activity are also being evaluated in larger multicenter trials [18-21].

## Conclusion

Laryngeal MCL is exceedingly rare. After an extensive literature search and to the best of our knowledge, this is the first reported case. MCL is very aggressive and has a poor prognosis. There currently is no consensus for a standardized treatment for this type of lymphoma. New therapeutic protocols, however, are under evaluation and may improve the outcome for patients.

## Consent

Written informed consent was obtained from the patient’s next of kin for publication of this manuscript and accompanying images. A copy of the written consent is available for review by the Editor-in-Chief of this journal.

## Abbreviations

CD, Cluster of differentiation; CT, Computed tomography; MCL, Mantle cell lymphoma; NHL, Non-Hodgkin lymphoma.

## Competing interests

The authors declare that they do not have any competing interests.

## Authors’ contributions

SN, AAB-B, MG, HM, SG, LE, MK and HE contributed equally to generating the experimental data. AAB-B performed the tracheotomy, laryngoscopy and laryngeal biopsy. SN was in charge of chemotherapeutic treatment and follow-up of the patient. AAB-B and SN wrote the article and performed the literature review. All authors read and approved the final manuscript.
